# The Need of Antimicrobial Stewardship in Post-Operative Infectious Complications of Abdominal Surgery

**DOI:** 10.1159/000526785

**Published:** 2022-10-21

**Authors:** Wilfried Obst, Torben Esser, Achim Jens Kaasch, Gernot Geginat, Frank Meyer, Roland S. Croner, Verena Keitel

**Affiliations:** ^a^Department of Gastroenterology, Hepatology and Infectious Diseases Medical Faculty of the Otto von Guericke University Magdeburg, Magdeburg, Germany; ^b^Institute of Medical Microbiology and Hospital Hygiene Medical Faculty of the Otto von Guericke University Magdeburg, Magdeburg, Germany; ^c^Department of General, Abdominal, Vascular and Transplant Surgery, Medical Faculty of the Otto von Guericke University Magdeburg, Magdeburg, Germany

**Keywords:** Post-operative infections, Antimicrobial stewardship, Intra-abdominal infections, Antibiotic stewardship, Abdominal surgery, Surgical site infections

## Abstract

**Background:**

Post-operative infection is a common complication following abdominal surgery. The two most common infections are secondary peritonitis and surgical site infections, which lead to increased perioperative morbidity, prolonged hospitalization, higher mortality rates, and increased treatment costs. In addition to surgical procedures, treatment is based on effective antibiotic therapy. Due to increasing antimicrobial resistance, the correct use of antimicrobials is becoming more complex. Many initiatives call for the implementation of an antimicrobial stewardship (AMS) programme to optimize anti-infective therapy. The review article summarizes current recommendations in anti-infective therapy of post-operative peritonitis and surgical site infections and highlights the importance of an AMS programme in abdominal surgery.

**Summary:**

Larger studies evaluating the benefit of AMS in abdominal surgery are lacking. However, national and international guidelines have formulated appropriate recommendations for the rational use of antibiotics in post-operative peritonitis and surgical site infections. The rate of post-operative infections can be significantly reduced by perioperative antibiotic prophylaxis. The increase in multidrug-resistant bacteria complicates anti-infective therapy for post-operative infections. Analysis of local susceptibility patterns helps choose an adequate empiric therapy. A high rate of extended-spectrum beta-lactamase-producing bacteria may necessitate the use of other reserve antibiotics in addition to carbapenems, which are approved for the treatment of complicated intra-abdominal infections. A key role for the AMS team is the subsequent de-escalation of antibiotic therapy which limits the use of unnecessary broad-spectrum antibiotics.

**Key Messages:**

The increase in multidrug-resistant bacteria poses challenges for abdominal surgery. Post-operative infections should be treated by an interdisciplinary team of surgeons and specialists for AMS.

## Introduction

Worldwide, about 4.95 million people die each year in connection with multidrug-resistant (MDR) bacteria. Intra-abdominal infections (IAIs) are the third most common origin. This alone accounts for about one million deaths each year caused by or associated with antibiotic-resistant pathogens [1]. Many international guidelines and law initiatives call for better use of antibiotics and promote “antimicrobial stewardship” (AMS). The aim of any AMS programme (ASP) is to optimize the use of antibiotics in order to [2, 3, 4, 5]

1. choose the optimal antibiotic
2. reduce the duration of antibiotic-associated therapy
3. reduce antibiotic side effects and infections with Clostridioides (C.) difficile
4. reduce length of hospital stay and overall treatment costs
5. reduce the emergence of antimicrobial resistance.

A number of meta-analyses have shown that these aims can at least partly be achieved for various infections, such as pneumonia, bloodstream infections, and urinary tract infections [6, 7, 8]. There is an ongoing debate whether ASP reduces infections by *C. difficile* and reduces antibiotic resistance [6, 8]. Very few studies directly addressed the outcomes of ASP in surgical patients. This is in contrast to the obvious need in abdominal surgery, where post-operative infection rates are reported between 8 and 20% [9, 10]. Corresponding initiatives and working groups on the rational use of antibiotics in abdominal surgery have therefore been established by national associations. The aim of this review article was to summarize current recommendations for anti-infective therapy in the two most common post-operative infections in abdominal surgery: post-operative peritonitis and surgical site infection (SSI) and to highlight the value of ASP in surgery.

## Risk Factors for Post-Operative Infection and Antibiotic Prophylaxis in Abdominal Surgery

It is important to complement an ASP with infection control measures [8]. Pre-operatively, patients who are at high risk for post-operative infections can be identified. Both patient-related and surgery-related factors play a role [11]. Important patient-related risk factors are, for example, pre-existing immunosuppression, chronic liver and kidney failure, diabetes as well as an abnormal body mass index in the form of obesity or malnutrition. Surgery-related factors can be caused pre-, intra-, and post-operatively. Relevant factors include emergency surgery, contaminated/infected wounds, or invasive devices. An overview of potential risk factors is shown in Table 1.

The use of adequate perioperative antibiotic prophylaxis (PAP) can reduce the rate of post-operative wound infections by up to 80% [12]. Both the World Health Organization and the European Centre for Disease Prevention and Control (ECDC) have published detailed guidelines for prevention of SSI, which describe not only PAP but also perioperative management [13, 14]. The cornerstones of (PAP) are

- selection and yearly review of PAP based on local pathogen and susceptibility data
- implementation plan for perioperative prophylaxis (assign responsibilities)
- administration of antibiotic prophylaxis 30–60 min before start of incision to obtain sufficient tissue levels
- single dose of antibiotic prophylaxis is preferred; subsequent doses should be given depending on the duration of the procedure and the half-life of the antibiotic, and if significant blood loss occurs during surgery
- no prolonged antibiotic administration over several days
- assessment of compliance to above rules on a regular base.

Prolonging antibiotic prophylaxis over several days has no effect on the post-operative SSI rate in patients undergoing colorectal surgery, which are at a particularly high risk of developing SSI [15]. Instead, prolonged administration can lead to a slightly increased rate of *C. difficile* infection and selection of MDR bacteria [13, 16, 17]. For abdominal surgery, however, the data are limited.

AMS can safely modify perioperative prophylaxis. For example, a study by Surat et al. [18] demonstrated that shift in prophylaxis from second generation cephalosporin to first generation cephalosporin was safe in cardiothoracic surgery patients. No increase of wound infections was observed.

Specific recommendations on PAP require data from clinical studies which are not available for many indications. Therefore, guidelines usually give general recommendations and advocate taking into account local pathogen and susceptibility data [19, 20]. A multidisciplinary team including infectious disease specialists, pharmacologist, and microbiologists should evaluate the in-house recommendations annually [13, 14]. For example, a table of recommendations of a tertiary university hospital based on the local resistance data is shown in Table 2. In general, like others [11, 21], for contaminated abdominal surgery, the use of a second-generation cephalosporin in combination with metronidazole in order to cover *Staphylococcus aureus*, Gram-negative bacteria and anaerobes is recommended. In the presence of contraindications (e.g., allergies), clindamycin plus gentamicin is an alternative choice. No PAP is required for uncomplicated clean surgery (e.g., in cholecystectomy without risk factors). For more extensive surgery with risk factors (e.g., pancreatic or liver resections), the frequent use of piperacillin/tazobactam due to frequent resistance of Gram-negative enteric bacteria to cefuroxime is established. The use of fluoroquinolones due to the high rate of potential serious side effects and available alternatives is no longer recommended [22].

Depending on local resistance rates, a common standard PAP does not cover MDR bacteria. The need of covering MDR bacteria in colonized patients is under debate. In general, coverage of MDR bacteria should follow an individual risk assessment to avoid the use of reserve antibiotics in PAP. However, initial data show that the rate of SSIs in patients with colonizing extended-spectrum beta-lactamase (ESBL)-producing bacteria can be further reduced [23, 24, 25]. A suggestion of how such an adjustment might look like is shown in Table 3 according to Eckmann et al. [26] and is based on expert opinion. But there is an ongoing and difficult debate about this issue. A differentiated consideration of benefits and risks in the use of reserve antibiotics in patients with MDR colonization must take place.

In general, a pre-operative screening for MDR bacteria can be recommended, based on patient-specific risk factors. Important criteria to be considered include: previous contact with a patients with MDR bacteria, need for dialysis, inpatient hospitalization (≥72 h) in the last 12 months, or patient coming from a high-prevalence country. The ECDC publishes annual reports for its member countries on the local resistance situation, which can be used for orientation [27]. In some countries, in-hospital MDR surveillance programmes and screening are mandatory [28].

## Post-Operative Infections

About 8–20% of abdominal surgery patients suffer from post-operative infections [9, 10], most notably complicated intra-abdominal infections (cIAIs) and SSIs, which are discussed below from the perspective of possible AMS interventions. Other post-operative infections account for only a small percentage of abdominal surgery patients. For example, the post-operative rate of *C. difficile* infection is generally less than 1.5%, nosocomial pneumonia about 1.1% and urinary tract infection 0.91% [29, 30, 31]. Nonetheless, these infections also lead to increased antibiotic consumption, longer hospital stays, and higher treatment costs which should also be addressed by a structured ASP as discussed above [6, 7, 8].

## Post-Operative IAIs

### Definition and Epidemiology

Secondary infections of the abdominal cavity are classified as either community acquired or health care IAIs [32] and account for about 80–90% of all IAIs. Post-operative/post-interventional peritonitis is a special form of secondary peritonitis. In contrast to community-acquired secondary peritonitis (e.g., acute perforated appendicitis), there is an increased rate of MDR bacteria, which is also associated with an increased mortality rate [32, 33]. This form of peritonitis is often caused by post-operative anastomotic insufficiencies/leakages or secondary organ perforation, where the organisms are spread beyond the hollow organs and lead to intra-abdominal abscesses and/or secondary peritonitis (cIAI).

Another special case is tertiary peritonitis. After surgical treatment has been completed, the infection persists in the abdominal cavity. This is usually caused by several patient-related risk factors, such as existing immunosuppression or alterations of the immune system (e.g., organ dysfunction; autoimmune diseases; malnutrition; sarcopenia; cachexia; hypalbuminaemia; advanced, long-lasting, or persisting malignancy; chemotherapy; [previous] therapy regimens with immunosuppressants; biologicals and/or anti-inflammatory drugs or even immune checkpoint inhibitors and others), which can prevent complete eradication of the infection. Tertiary peritonitis is characterized by an increased rate of MDR bacteria and often presents a therapeutic challenge. A gradual transition from secondary to tertiary peritonitis is possible [32].

Current data on the incidence and mortality of post-operative IAI are limited, so that the current situation cannot be adequately assessed. However, in older reports, IAIs are responsible for about 25–30% of septicaemias and septic shock, and about 150,000 patients are treated with these indications in Germany annually [33, 34]. In a more recent retrospective analysis, the overall mortality of post-operative peritonitis was 26%, with the highest mortality rate of 40% occurring in duodenal perforations [35].

Microbiologically, there is often a polymicrobial infection with facultative aerobic enteric bacteria (e.g., *Escherichia coli*, *Klebsiella* spp., *Citrobacter* spp., *Enterococcus* spp.) and obligate anaerobic bacteria (e.g., *Bacteroides* spp., *Peptostreptococcus* spp., *Clostridium* spp.). In addition, intra-abdominal fungal infection with *Candida* spp. are found in about 50% of cases [11, 33, 35]. Depending on the localization, characteristic microbial spectra can usually be detected. Leaks in the upper gastrointestinal tract (stomach, duodenum, proximal jejunum) usually cause infections with Gram-positive and Gram-negative facultative aerobic bacteria, such as staphylococci and *E. coli.* Leaks in the mid or distal small intestine often lead to purely Gram-negative bacterial infections, which can be both facultative and obligate anaerobic (e.g., *Bacteriodes fragilis*). Insufficiencies in the colon or rectum are mainly characterized by anaerobes (e.g., *C.* spp.) and *Enterococci* in addition to Gram-negative bacteria [11, 36].

In contrast to community-acquired IAIs, MDR bacteria must also be considered in post-operative cIAIs. These can either be acquired in hospital or already present at the time of admission, especially Gram-negative enteric bacteria, for example, *E. coli*, *K.* spp., with ESBLs or carbapenemases but also *Enterococci*, particularly vancomycin-resistant *E. faecium* and *C.* spp. are present frequently [11, 35]. Therapeutic options for Gram-negative bacteria producing CPE are scarce. In the case of cIAI, these are mainly *Enterobacterales* (*Klebsiella pneumonia*, *E. coli)*, for which only reserve antibiotics are effective [35, 37]. Other MDR bacteria with carbapenem resistance comprise *Pseudomonas aeruginosa* and *Acinetobacter baumannii*, which, however, still rarely occur in post-operative cIAI [26].

The increased mortality due to infection with MDR bacteria does not result from an increased virulence of the pathogens but rather from a lack of response to the initial empiric antibiotic therapy [33, 37]. Screening for MDR bacteria can guide empiric antibiotic therapy. A study from 2016 showed that about 9.5% of the admitted patients in Germany were colonized with an ESBL-producing bacterium [38]. Early detection of a colonization with MDR bacteria could provide the following advantages:

- reduction of SSI by adjusting the perioperative choice of antibiotics,
- improving the choice of empiric antibiotic for post-operative infections, and
- preventing the in-hospital spread of MDR-bacteria by early implementation of infection control measures.

### Antibiotic Therapy of Post-Operative Peritonitis

Treatment of post-operative secondary peritonitis should always be interdisciplinary, involving a team of surgeons, intensive care specialists, and the AMS team. The most important procedure remains the surgical/interventional treatment of the underlying cause (anastomotic leakage/hollow organ perforation). The surgical concept of “source control” consisting of debridement, removal of infected devices, drainage of purulent cavities, and decompression of the abdominal cavity contributes significantly to reducing mortality. Current developments of this established process are increasingly focussing on “damage-control surgery,” where the source of infection is treated first, and the correct anatomy is restored in a second step after stabilization of the patient [39].

When surgical “source control” can be achieved, the duration of antibiotic therapy can be reduced. This should be a focus of ASP. In the study by Sawyer et al. [40], the outcome of a 4-day fixed-duration antibiotic therapy was similar to a longer, on average 8-day course of antibiotics that extended until after the resolution of physiological abnormalities. In addition to prompt surgical therapy, the rapid initiation of an effective antibiotic therapy is crucial for patient outcome in critical cases [37].

Empiric antibiotic therapy should be started immediately, especially to reduce progression to sepsis, if not already present. In sepsis, even a moderate delay in antimicrobial therapy is associated with increased mortality [41]. The choice of the initial antibiotic therapy should follow current guidelines, take local susceptibility data into account, and reflect the patient's risk factors (known colonization with MDR bacteria, allergies, renal function).

In contrast to community-acquired secondary peritonitis, empiric therapy of health care intra-abdominal cIAI should also cover ESBL bacteria. According to the results of the MERINO study, piperacillin/tazobactam was inferior to meropenem in bacteraemia with ESBL-producing *E. coli* or *Klebsiella pneumoniae*, which were measured susceptible to both agents [42]. Accordingly, carbapenems should be used preferentially in infections with ESBL-producing bacteria [43]. According to German guidelines, tigecycline, ceftolozane/tazobactam (in combination with metronidazole), and ceftazidime/avibactam, which are all approved for cIAI, could be used as 2nd line therapy. In general, the routine prescription of the new cephalosporin-beta-lactamase inhibitor and carbapenem-beta-lactamase inhibitor combinations as well as the new siderophore cephalosporin cefiderocol [44] should be avoided. This strategy follows the current recommendations of the European Society of Clinical Microbiology and Infectious Diseases. Their use is restricted to otherwise non-susceptible pathogens [45].

Whether coverage for VRE should be included in empiric therapy is controversial. If VRE colonization is known pre-operatively, the combination with linezolid can be considered [26, 43]. If the patient has risk factors for a severe course (immunosuppression, high risk of endocarditis, abdominal sepsis), we recommend to add linezolid. In contrast, empiric therapy with antifungal agents has no effect on mortality and is not recommended [43, 46]. In general, microbiological findings of *Enterococcus* spp. and/or *Candida* spp. should be critically discussed within the AMS team. It is often not entirely clear to what extent these pathogens are responsible for the pathogenesis of cIAI or whether representing incidental findings of colonization. An overview of the initial therapy of post-operative peritonitis is shown in Table 4 and an overview of the currently possible antibiotic therapies for multi-resistant pathogens is shown in Table 5.

With availability of the microbiological susceptibility testing, de-escalation of antibiotic therapy to an agent with a narrower spectrum should be done to limit the use of empiric broad spectrum antibiotics. Septic patients should be treated according to the current sepsis guidelines [47]. However, this should be done under close monitoring, as the results of microbiological samples of the abdomen may also not represent all relevant pathogens. In septic patients, therapeutic drug monitoring in combination with a “drug interaction stewardship” improves the overall therapeutic quality. As published, especially in critically ill patients, serum antibiotic levels were outside of the recommended range in 51% of cases, necessitating dose adjustments [48].

In a typical AMS visit, the above points should be discussed within the framework of the local ASP in an interdisciplinary team. There is a need for weekly visits of the AMS team to the intensive care units and surgical wards. Complicated cases are discussed here on an interdisciplinary basis. The most important topics need to be covered during the visits are accurate diagnosis of an infection, shifting form broad-spectrum to small-spectrum antibiotics and limiting the duration of the antibiotic therapy. The effect of AMS visits has been demonstrated in some studies for surgical wards. With the same treatment outcome, the consumption of antibiotics, especially with broad spectrum, decreased up to 18.3%, while the consumption of penicillins with narrow spectrum increased up to 89.9%. At the same time, not only the duration of antibiotic therapy but also the costs were reduced [49]. However, only regular AMS visits seem to have a lasting effect on the prescribing habits on surgical wards [50].

## Surgical Site Infections

### Epidemiology and Classification

Besides post-operative IAIs, SSIs are the second major group of post-operative infections in abdominal surgery. These are defined by the occurrence of a wound infection within 30 days after surgery and are classified into 3 severity levels [51, 52]:

1. superficial wound infections (grade 1),
2. seep wound infections (grade 2), and
3. infections of organs and body cavities in the surgical area (grade 3).

According to ECDC data, colorectal surgery has the highest rate of SSI across operations, with an incidence of 10.1 per 100 operations. The proportion of deep infections or involvement of organs and body cavities is particularly high and occurs in 61% of cases [53]. Although advances have been made in infection control practices, surgical technique, and availability of antimicrobial prophylaxis, SSIs remain a considerable cause of morbidity, prolonged hospitalization, and death [52].

Mixed bacterial infections cause the majority of SSIs, while viral and fungal infections are rare. Most SSIs in abdominal surgery are caused by *E. coli*, *Enterococcus* spp., *Bacteroides* spp. and less frequently by *Staphylococci* and *Streptococci.* Post-operatively, the proportion of potential MDR bacteria in particular increases. Here, Gram-negative *Enterobacterales* continue to form the largest proportion of isolates, which are followed by *Enterococci* [51, 53]. The number of infections caused by fungi is low at 2.7/100 per SSI but higher than in other surgical procedures [51].

### Infection Prevention

Perioperative interventions selected specifically for the particular patient can reduce the rate of SSIs. There are many recommendations and published reviews that address this issue in more detail [54]. From the perspective of AMS, PAP (see section on PAP), which has already been discussed in detail, and antiseptic treatments play a central role in prevention of SSIs. Intra-operative subcutaneous wound irrigation can additionally reduce the rate of SSI. In a randomized controlled trial, Strobel et al. [55] showed that the rate of superficial SSIs can be significantly reduced by irrigating the wound with 0.04% polyhexanide solution before wound closure compared with 0.9% saline. Similar results were obtained by Gotzok et al. [56] after temporary loop ileostomy. Accordingly, antiseptic treatment is listed as a preventive measure for post-operative wound infections in the current recommendations of the RKI [51].

### Therapy

Surgical treatment is also the basis for the treatment of SSIs. Source control involves drainage of abscesses or infected fluid collections, debridement of necrotic or infected tissues, and definitive control of the source of contamination. From the perspective of the AMS, systemic antibiotic drugs should be used in a stage-adapted manner. Uncomplicated grade 1 or 2 SSIs can usually be treated surgically and with the use of antiseptic solutions without the use of antibiotics. Microbiological sampling can identify organisms and corresponding susceptibility patterns at an early stage, which guide therapy, if the infection progresses. Exceptions are phlegmons or erysipelas, which can spread from the wounds and have to be treated with antibiotics immediately. In contrast to grade 1 and 2 infections, grade 3 SSI always require a systemic treatment [51, 52, 54]. The anti-infective treatment is analogous to the procedure for post-operative peritonitis as mentioned above. Cornerstones are

1. select an empiric therapy based on risk factors for the presence of MDR bacteria as well as knowledge of local pathogen and susceptibility data
2. focus on antimicrobial therapy after microbiology results are available
3. avoid unnecessary prolonged antibiotic therapy after surgical control of the focus.

## Conclusion

The increase in multi-resistant bacteria also poses a challenge to abdominal surgery. An interdisciplinary team of surgeons and AMS specialists should work together to treat post-operative infectious complications. In addition to surgical therapy, anti-infective therapy is also crucial for the success of the treatment. In this context, principles of AMS should guide antibiotic therapy in post-operative infections in abdominal surgery.

## Conflict of Interest Statement

All authors declare that they have no competing interests or conflicts of interest. Wilfried Obst received a one-time fee for participating in an advisory board for Gilead. Verena Keitel received speaker's reimbursement from Albireo, Falk Foundation, CSL Behring, AbbVie, Intercept, and Gilead and a one-time fee for participating in an advisory board for Astra Zeneca.

## Funding Sources

There was no financial support for this work.

## Author Contributions

Wilfried Obst received the invitation and drafted the literature review, the outline, and the manuscript. Torben Esser made a substantial contribution to the SSI section. Achim Jens Kaasch, Gernot Geginat, Frank Meyer, Roland Croner, and Verena Keitel made corrections to the manuscript and substantively contributed content and ideas to improve the review article.

## Figures and Tables

**Table 1:** Risk factors for post-operative infections (modified from Hagel and Scheuerlein [11])

Risk factors for post-operative complications	
patient related	surgery related
Age	*Pre-operative*
Alcohol abuse	Emergency surgery
Implanted biomaterial	Previous radiation therapy
Compromised immunity/immunosuppression	Prolonged pre-operative hospitalization
Chronic heart failure	*Intra-operative*
Diabetes	Contaminated/infected surgical field
Dialysis	Decreased oxygen saturation
Drug abuse	Diathermy
Fever (within 1 week pre-operatively)	Duration of surgery >2 h
Liver cirrhosis	Hypothermia
Malnutrition	More than one surgical intervention
MSSA/MRSA carrier	Prolonged duration of anaesthesia
Neuropathy	Wound contamination with *Enterococcus*
	spp., *Enterobacterales, Bacteroides fragilis*
Obesity	
Previous antibiotic treatment	*Post-operative*
Previous other infection	Drainage device >3 days
Poor general condition	Invasive device, for example, urinary
Radiation	catheter, chest drain, nasal tube, central
Sarcopenia	venous catheter
Smoking	Respiratory sepsis
Stoma	

**Table 2:** Recommendations for PAP at the University Hospital of Magdeburg (modified from Bratzler et al. [21])

PAP		

Surgery	antibiotic 1st choice	alternative in case of contraindication/allergies
*Appendectomy*	Cefuroxime 1.5 g + metronidazole 500 mg	Clindamycin 600 mg + gentamicin 5 mg/kg

*Hernia surgery*		
Without mesh	No antibiotic prophylaxis	
With mesh	Cefazolin 2 g	Vancomycin 1–1.5 g

*Colorectal surgery*		
Without risk factors	Cefuroxime 1.5 g + metronidazole 500 mg	Clindamycin 600 mg + gentamicin 5 mg/kg
With risk factors	Piperacillin/tazobactam 4.5 g + gentamicin 5 mg/kg	

*Laparoscopic cholecystectomy*		
Without risk factors	No antibiotic prophylaxis	
With risk factors	Cefuroxime 1.5 g + metronidazole 500 mg	Clindamycin 600 mg + gentamicin 5 mg/kg
*Laparoscopic cyst surgery (no suspicion of echinococcosis)*	No antibiotic prophylaxis	

*Liver or pancreas resection*		
Without risk factors	Cefuroxime 1.5 g + metronidazole 500 mg	Clindamycin 600 mg + gentamicin 5 mg/kg
With risk factors	Piperacillin/tazobac. 4.5 g	

*Liver transplantation*	Piperacillin/tazobac. 4.5 g	Clindamycin 600 mg + gentamicin 5 mg/kg

*Emergency interventions (ileus, perforation)*	Cefuroxime 1.5 g + metronidazole 500 mg	Clindamycin 600 mg + gentamicin 5 mg/kg

*Proctology interventions, goitre surgery, soft tissue tumours without hollow organ involvement*	No antibiotic prophylaxis	

**Table 3:** Proposal for PAP in patients colonized with MDR bacteria, modified according to Eckmann et al. [26]

PAP in case of MDR bacteria colonization	
MDR bacteria	antibiotics
ESBL *E. coli*	Ertapenem 1 × 1 g
	Tigecycline 1 × 100 mg
ESBL *Klebsiella* spp.	Ertapenem 1 × 1 g
	Tigecycline 1 × 100 mg
Carbapenem-resistant *Pseudomonas* spp.	Ceftolozane/tazobactam 1 × 1.5 g
	Ceftazidime/tvibactam 1 × 2.5 g
Carbapenem-resistant *Acinetobacter* spp.	Tigecycline 1 × 100 mg
Carbapenem-resistant *Klebsiella* spp. (KPC)	Ceftazidime/avibactam 1 × 2.5 g
	Tigecycline 1 × 100 mg

However, the use should not be routine and should only be decided individually.

**Table 4:** Recommendation for empiric antibiotic therapy of post-operative peritonitis according to German Paul-Ehrlich-Society for infection therapy [39]

Empiric initial therapy of post-operative peritonitis			
possible bacteria	antibiotics	dosage	therapy duration
*Enterobacterales* (incl. ESBL-formers)	Tigecycline	2 × 0.05–0.1 g* 3 × 2 g (+2 × 0.6 g)	7–10 days
*Enterococci* (incl. VRE)	Meropenem (+linezolid)	3 × 1 g (+2 × 0.6 g)	
Anaerobic	Imipenem (+linezolid)	3 × 1.5–3 g + 3 × 0.5 g (+2 × 0.6 g)	
*Pseudomonas* spp.	Ceftolozane/tazobactam + metronidazole (+linezolid)	3 × 2.5 g + 3 × 0.5 g	
*Staphylococci* (incl. MRSA)	Ceftazidime/avibactam + metronidazole (+linezolid)	(+2 × 0.6 g)	
	Fosfomycin (no monotherapy)	3 × 4–8 g	

* Loading dose for tigecycline 0.1 g.

**Table 5:** Recommendation for antibiotic therapy of post-operative peritonitis for MDR bacteria according to the German Paul-Ehrlich-Society for infection therapy [39]

Antibiotic therapy of secondary peritonitis in case of MDR bacteria	
MDR bacteria	recommendation
ESBL-producing *Enterobacterales* (e.g., *E. coli, Klebsiella* spp.)	Tigecycline
	Ceftolozane/tazobactam
	Ceftazidime/avibactam
	Imipenem
	Meropenem
	Ertapenem

Carbapenem-resistant *Enterobacterales* (e.g., *E. coli, Klebsiella* spp.)	Fosfomycin (no monotherapy) Tigecycline
	Colistin
	Ceftazidime/avibactam
	Meropenem (high dose)

Carbapenem-resistant *Pseudomonas* spp. with fluorchinolone-resistance	Ceftolozane/tazobactam
	Ceftazidime/avibactam
	Colistin

Carbapenem-resistant	Tigecycline
*Acinetobacter* spp.	Colistin

Vancomycin-resistant *E. faecium* or *E. faecalis* (AER)	Tigecycline
	Linezolid (in combination)

Methicillin-resistant *Staphylococcus aureus* (MRSA)	Tigecycline
	Linezolid (in combination)
	Vancomycin (in combination)

Vancomycin and linezolid should be used in combination with other antibiotic substances to treat Gram-negative bacteria as well.
